# Enhanced Light Extraction from Organic Light-Emitting Diodes with Micro-Nano Hybrid Structure

**DOI:** 10.3390/nano12081266

**Published:** 2022-04-08

**Authors:** Eun-Jeong Bae, Shin-Woo Kang, Geun-Su Choi, Eun-Bi Jang, Dong-Hyun Baek, Byeong-Kwon Ju, Young-Wook Park

**Affiliations:** 1 Nano and Organic-Electronics Laboratory, Department of Display and Semiconductor Engineering, Sun Moon University, Asan 31460, Korea; baeej2@sunmoon.ac.kr (E.-J.B.); newoosw@korea.ac.kr (S.-W.K.); crs4964@sunmoon.ac.kr (G.-S.C.); kksk0428@sunmoon.ac.kr (E.-B.J.); 2 Display and Nanosystem Laboratory, Department of Electrical Engineering, Korea University, Seoul 02841, Korea; 3 Center for Next Generation Semiconductor Technology, Department of Display and Semiconductor Engineering, Sun Moon University, Asan 31460, Korea

**Keywords:** organic light-emitting diodes, light extraction, reactive ion etching, mico-nano hybrid structure

## Abstract

In this study, an external light extraction layer with a micro-nano hybrid structure was applied to improve the external light extraction efficiency of organic light-emitting diodes (OLEDs). A reactive ion-etching (RIE) process, using O_2_ and CHF_3_ plasma, was performed on the surface of the micro-scale pattern to form micro-nano hybrid structures. According to the results of this study, the nanostructures formed by the treatment of O_2_ and CHF_3_ were different, and the efficiency according to the structures was analyzed experimentally and theoretically. As a result, the OLED, to which the micro-nano hybrid structure, manufactured through a simple process, is applied, improved the external light extraction efficiency by up to 38%, and an extended viewing angle profile was obtained. Additionally, an effective method for enhancing the out-coupling efficiency of OLEDs was presented by optimizing the micro-nano hybrid structure according to process conditions.

## 1. Introduction

Organic light-emitting diodes (OLEDs) are attracting attention as the next-generation technology in displays and light sources due to their low power consumption, high color purity, gamut, and applicability to flexible display devices [1,2,3,4]. They have also been studied for decades because of their potential in fields such as flexible, wearable, and rollable displays [5,6,7,8]. However, according to recent research results, the OLEDs have to overcome the problems of relatively low power efficiency and lifespan for next-generation displays and light sources [9]. The internal quantum efficiency (IQE) of OLEDs was achieved at 100% [10,11], but the quantum efficiency of the OLEDs was 20%; thus, the light generated inside of 80% cannot be emitted to the outside. To solve this problem, light extraction techniques are being actively studied [12]. The light extraction technology can be divided into an internal light extraction technology that can reduce the loss due to the waveguide effect between the substrate and the organic thin film, and an external light extraction technology that reduces the light loss due to the total reflection effect between the substrate and the air interface. Internal light extraction techniques that change the internal structure of a device include a low-refractive-index grid structure, an internal scattering layer structure, and a diffraction grating structure [13,14,15,16,17]. However, as the internal light extraction technology affects the internal structure of the device, a high-level technology that does not change the electrical characteristics of the device is required [18]. The external light extraction technology extracts light by modifying the optical structure of a flat glass substrate by changing the total reflection angle of the light incident on the external light extraction layer. Representative technologies reported so far include external light-scattering layer technology, micro-nano structure, and MLA. As detailed technologies, external light extraction layer implementation using polymer-silica, flexible OLEDs using the micro-lens array (MLA), and a light-scattering layer having a porous or bowl structure have been reported [19,20,21,22,23,24,25,26]. When an external light scattering layer is applied to a device, the radiation pattern exhibits a Lambertian distribution, which has the advantage of minimizing changes in luminance and color coordinates relative to the viewing angle [27]. However, most of them require a solution process with a complex chemical synthesis process and a long process time or require expensive materials and equipment. Moreover, in the case of a light extraction structure having a grating structure, the efficiency is increased because the diffraction condition is determined according to the period and a wavelength of light, but spectral distortion occurs at a specific wavelength other than the vertical direction [28]. However, the lens can be easily processed with a large area and at a low cost. In the case of using MLA, the angle range in which incident light can be extracted is increased by avoiding the total reflection due to the curved structure formed on the substrate [29]. The best way to extract the substrate mode is to apply a hemispherical lens. The lens diameter is much smaller than the emission area of OLEDs. The emission area does not depend greatly on the lens diameter within a range larger than the wavelength of light [30]. The research published so far has studied MLA and external scattering layers of various geometries such as hemispherical, cylindrical, pyramidal, and square made of polyethylene terephthalate and polymethyl-methacrylate materials [31,32]. However, the effect of light out-coupling on the OLEDs, to which micro-nano hybrid patterns are applied by integrating nano-scale patterns into the MLA and the external light extraction layers, has not yet been studied.

In this study, an external light extraction layer with a micro-nano hybrid structure was applied to improve the external light extraction efficiency of OLEDs. The micro-nano hybrid structure was fabricated through reactive ion etching (RIE) on the hexagonally packed 75 μm MLA surface. In the RIE process, irregular nanostructures of different sizes were formed on the micro-scale pattern surface by controlling the processing time using O_2_ and CHF_3_ plasma. The improvement of light out-coupling efficiency was analyzed experimentally and theoretically by integrating an external light extraction layer with a nanostructure with the OLEDs. The OLEDs with a micro-nano hybrid-structured MLA demonstrated highly improved external quantum efficiency (EQE), up to 38%, compared to the bare OLEDs; it also demonstrated a 12% improved EQE compared to the nonpatterned MLA-attached OLEDs. This study proves that the external light extraction efficiency is improved by applying the micro-nano hybrid structure, fabricated through a simple process, to OLEDs.

## 2. Experimental Section

### 2.1. RIE Process of External Light Extraction Layer

**Figure 1:**
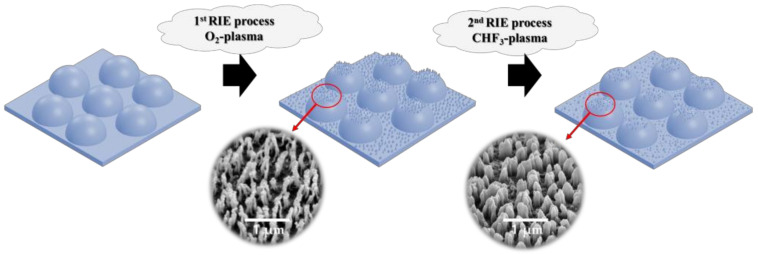
RIE process.

The RIE process was performed on the surface of the external light extraction layer with a pattern to implement a micro-nano hybrid structure. The external light extraction layer is used for the hexagonally packed 75 μm hemisphere pattern. The nanostructures on the surface of the micro-scale external light extraction layer were formed through the O_2_ and CHF_3_ plasma of the RIE system. As shown in Figure 1, after the O_2_ plasma treatment, the CHF_3_ plasma was secondly treated. The plasma process power was fixed at 200 W, and the process vacuum was performed at 32 mTorr. The different nanostructures were formed on the micro-patterns according to the plasma process time of O_2_ and CHF_3_. Eleven conditions with the RIE process were applied to MLA with different O_2_ and CHF_3_ gas plasma times from 50–400 s.

### 2.2. Fabrication of Organic Light-Emitting Diodes

**Figure 2:**
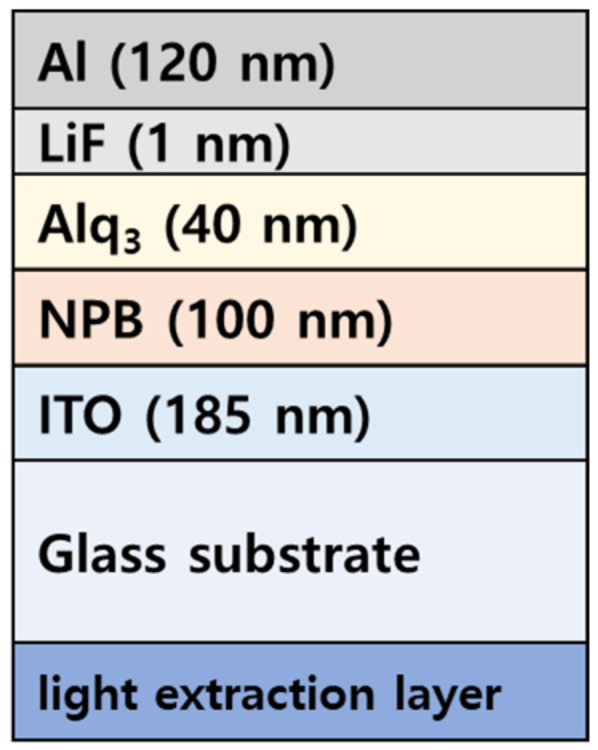
OLEDs device.

The soda-lime glass coated with 185 nm indium tin oxide (ITO) was cleaned with acetone, methanol, and deionized water using an ultrasonic cleaner for 15 min, respectively. The cleaned glass substrate was dried at 120 °C for 1 h in a dry oven. The light-emitting region of OLEDs was defined by a photoresist (AZ 601 GXR, AZ Electronic Materials CO., Ltd., Hsinchu, Taiwan) with a circle area of about 30.68 mm^2^ during the photolithography. The prepared substrate was treated with UV ozone (UVC-300, Omniscience, Gyeonggi-do, Korea) and O_2_ plasma (CUTE, Femto Science Co., Hwaseong-si, Korea) to reduce the driving voltage by removing residual contaminants and adjusting the work function of the anode. In Figure 2, the fabricated device is fluorescent, with OLEDs having a multilayer structure of the hole injection layer (HIL), hole transport layer (HTL), emitting layer (EML), the electron transport layer (ETL), and electron injection layer (EIL). The 185 nm of ITO was used as an anode, and 100 nm of N,N′-Bis(naphthalen-1-yl)-N,N′-bis(phenyl)benzidine (NPB) was used as an HTL 40 nm of Tris(8-hydroxyquinoline). Aluminum (Alq_3_) was used as an emission-electron transport layer, 1 nm of lithium fluoride (LiF) was used as an EIL, and 120 nm of aluminum (Al) was used as the cathode. All organic and metals used in this study were rotationally deposited in a high vacuum. The deposition rate of organic and metal was up to 1 Å/s and 3 Å/s, respectively. The thickness of the thin film was controlled at 6 MHz (QCM, Phillip Technologies, Greenville, SC, USA), with a thin film deposition controller (IQM-233, INFICON, Bad Ragaz, Switzerland). [33] A total of 13 OLED devices without and with nanopatterned/non-patterned MLA were fabricated and evaluated.

### 2.3. Characterization and Measurement

The pattern of the external light extraction layer was analyzed with a field emission scanning electron microscope (FE-SEM, F-4800, Hitachi, Japan). To analyze the light extraction characteristics of each micro-nano hybrid structure formed in the external light extraction layer, an optically clear adhesive (OCA) film was attached to the glass bottom/emitting surface of the manufactured OLEDs. The electroluminescent (EL) characteristics of the OLEDs with an external light extraction layer were measured using a spectroradiometer (CS-2000, Konica Minolta Co. Ltd., Chiyoda, Tokyo, Japan) and a source meter (Keithley-2410, Tektronix, Beaverton, OR, USA). The EL characteristics, including the viewing angle, were measured in a dark box with CS-2000 assuming a Lambertian light source of OLEDs, and the area of measurement spot was 0.0314 mm^2^. The viewing angle characteristics of the fabricated device were measured by rotating the device by 10° from 0° to 70° using a rotation stage. The EQE was recalculated by applying viewing angle characteristics (Figure S1 and Table S1).

## 3. Result and Discussion

### 3.1. Micro-Nano Hybrid Structure of Extraction Layers

**Figure 3:**
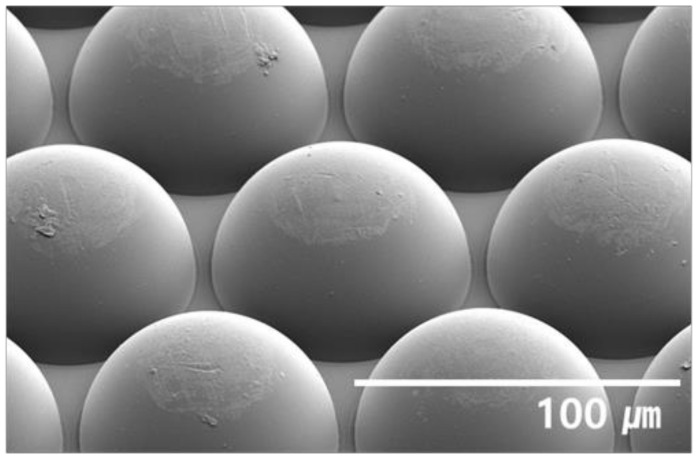
A 45° tilted SEM image of hexagonally packed hemisphere MLA for light extraction layer.

Figure 3 shows an external light extraction layer of the hexagonally packed 75 μm hemisphere MLA.

**Figure 4:**
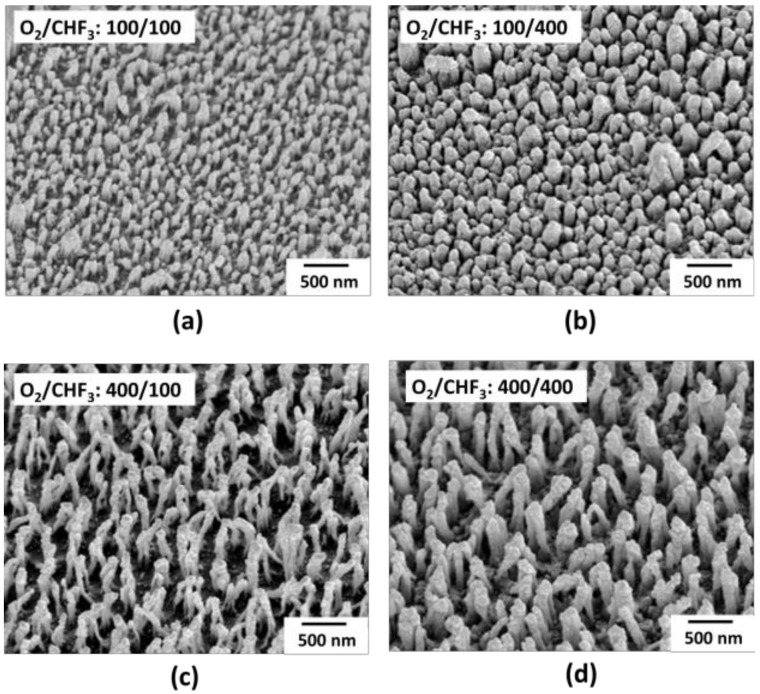
SEM image of MLA surface with different RIE process (**a**) O_2_ 100 s, CHF_3_ 100 s, (**b**) O_2_ 100 s, CHF_3_ 400 s, (**c**) O_2_ 400 s, CHF_3_ 100 s, and (**d**) O_2_ 400 s andCHF_3_ 400.

The RIE process was performed on the surface of the light extraction layer with micro-scale patterns. In the RIE process, the process times of O_2_ and CHF_3_ were adjusted to 50 s and 400 s, and the surface structure of the pattern according to the process times was analyzed. The representative and distinguishable four conditions of RIE patterned MLAs were presented, from small-sized to bigger ones. Figure 4 is the SEM images of the measured surface changes caused by the RIE process of the hemisphere MLA. In Figure 4, irregular nanostructures were formed on top of micro-patterns after the RIE process.

Comparing Figure 4a–d, it is evident that with a longer O_2_ process time, a deeper surface structure of the hemisphere MLA was etched, and deeper irregular nanostructured pillars were formed. The nanostructured pillars formed had a height between 140–180 nm when treated with O_2_ for 100 s. The height of the nanostructured pillars increased up to 375 nm in Figure 4c,d when treated with O_2_ for 400 s. Comparing Figure 4a–d, it is evident that as the CHF_3_ process time increased, the nanostructures formed by the O_2_ process were condensed. In Figure 4a,c, when treated with CHF_3_ for 100 s, the width of the nanostructures formed was less than 100 nm. On the other hand, as shown in Figure 4c,d, when treated with CHF_3_ for 400 s, the width of the nanostructures formed increased to approximately 150 nm. As the O_2_ process time increased, the surface of MLA was etched deeper, and the height of the nanopillars increased. Moreover, as the CHF_3_ process time increased, the nanopillars were condensed by fluorine, and the width increased [34].

### 3.2. Characteristics of OLEDs with External Light Extraction Layer of Micro-Nano Hybrid Structure

The external light extraction layer formed by the RIE process was attached to the glass bottom/emitting surface of the fabricated OLEDs with OCA, and the EL characteristics of the device were measured. Reference is a bare OLEDs in which an external light extraction layer is not applied. The characteristics of the external light extraction layer without the RIE process applied to it and the micro-nano hybrid structure formed according to the O_2_ and CHF_3_ process time were evaluated.

**Figure 5:**
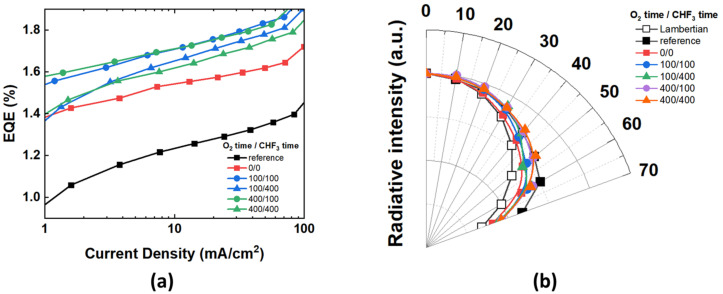
EL characteristics of OLEDs devices without and with nanopatterned/non-patterned MLA (**a**) current density-EQE graph, and (**b**) the angular intensity distribution.

Figure 5 shows the current density–EQE characteristics of the fabricated OLEDs without and with nanopatterned/non-patterned MLA. The EL characteristics are analyzed at a current density of 20 mA/cm^2^. The EQE of the reference device is 1.28%. All the devices with an external light extraction layer demonstrate an improvement in EQE compared to the reference device. In Figure 5a, the EQE of the 0/0 without the nanostructure was 1.57%, which increased by 23% compared to the reference. The efficiency was improved due to the light scattering induced by the applied micro-scale hemisphere pattern. On the while, all the devices with micro-nano hybrid structure showed greater improvement than the references. Furthermore, compared to the reference device, the micro-nano hybrid structure demonstrated a greater improvement in efficiency than the 0/0 (bare micro-scale hemisphere pattern). The EQE of the 100/100 and 100/400 was 1.77% and 1.71%, respectively, which improved by 38% and 33% compared to the reference. The EQE of 400/100 and 400/400 was 1.76% and 1.68%, respectively, which improved by 38% and 31% compared to the reference. Additionally, the EQE of the 100/100, 100/400, 400/100, and 400/400 were 13%, 9%, 12%, and 7% higher, respectively, compared to 0/0, where the nanopattern was not formed. The nanopatterned surface made the trapped light to be extracted to air by changing the incident angle of the light [32,35]. To analyze the viewing angle characteristics shown in Figure 5b, the device was measured from angles 0° to 70° (with 10° increments). The EL intensity for each device at various viewing angles was measured and normalized based on an intensity of 0°. In the viewing angle characteristics, all devices showed a wider profile than the Lambertian light source. The emission profile of the device with the hemisphere MLA applied for shows that it is closer to the Lambertian distribution than the reference device. The viewing angle distribution of 100/100, 100/400, 400/100, and 400/400 with the micro-nano hybrid structure was reduced by up to 3% compared to the reference device but increased by up to 10% compared to the 0/0 without a nanopattern. The device with the hemisphere MLA has applied a relatively wide radiation pattern at 400/100 and 400/400. The EQE characteristic was affected by the CHF_3_ process time, but the viewing angle characteristic was affected by the radiation profile according to the O_2_ process time. As mentioned previously, we demonstrated that the longer the O_2_ process time, the larger the height of the nanostructures formed. (Figure S2) In this light extraction structure, the micro-scale MLA pattern extracts the substrate mode light, and the nanopattern is used as a light-scattering layer to extract light into the air. This extracts the light that cannot be extracted only with the nanopattern by changing the angle of the incident light by the micro-scale curvature [24]. It can be confirmed that the viewing angle can be adjusted without spectral distortion through the process used in this study (Figures S3 and S4) [35].

**Figure 6:**
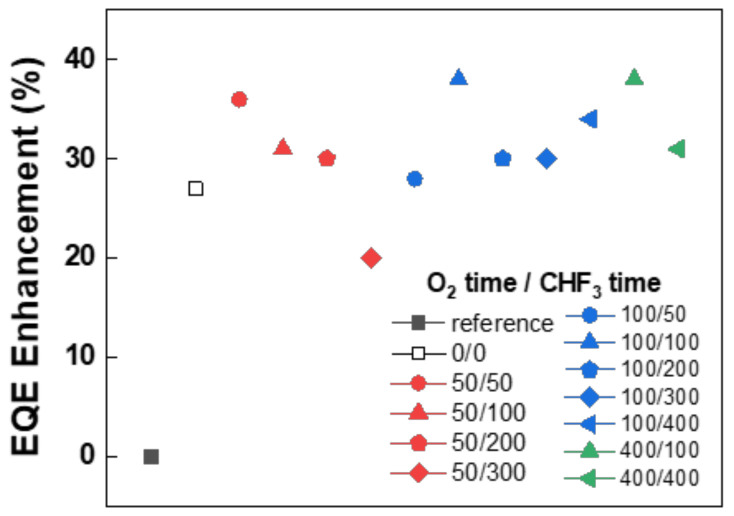
EQE enhancement according to RIE plasma process time compared to the bare OLEDs.

Figure 6 shows the EQE enhancement of all the fabricated devices. The pattern image of all fabricated conditions is in Figure S5. The EQE enhancement was more affected by the CHF_3_ process time than the O_2_ process time. When the CHF_3_ process time was the same, the shorter O_2_ process time demonstrated a higher efficiency improvement, but the difference in EQE enhancement compared to the reference according to the change in O_2_ process time was within 10%. Whereas, when the O_2_ process time was the same, the EQE enhancement was different up to 16% compared to the reference as the CHF_3_ process increased. Among all devices, the 100/100 light extraction efficiency demonstrated a significant improvement of 38%, and it can be observed that the height of the nanostructure is about 180 nm, and the width is 100 nm, which is the most suitable structure for light extraction.

## 4. Conclusions

In summary, the light extraction efficiency of OLEDs was improved by fabricating a micro-nano hybrid structure using a simple process. A nanostructure was formed by performing the RIE process on the micro-patterned surface of the hexagonally packed hemisphere MLA. The fabricated nanostructures showed different shapes and density depending on the plasma process time of O_2_ and CHF_3_. Following the gas plasma time increases, the height and the width of nanopillars also increased. The EL efficiencies demonstrated significant dependence following the shape and density change of the nanostructure. Thus, the OLEDs with micro-nano hybrid-structured MLA demonstrated highly improved EQE, up to 38%, compared to the bare OLEDs; it also demonstrated a 12% improved EQE compared to the non-patterned MLA-attached OLEDs. The micropattern extracts the substrate mode, light, and the nanopattern is used as a light-scattering layer to extract light into the air. From this result, it can be confirmed that the viewing angle of the OLEDs can be adjusted. By applying and controlling the gas plasma process on the MLA, we easily and successfully demonstrated the micro-nano hybrid-structured light extraction layer and improved light extraction characteristics. The results demonstrate an effective methodology for improving the out-coupling efficiency of OLEDs by implementing a micro-nano hybrid structure through a simple process. It is also expected to provide another opportunity to achieve highly efficient OLEDs.

## Data Availability

Not applicable.

## Supplementary Materials

The following supporting information can be downloaded at: https://www.mdpi.com/article/10.3390/nano12081266/s1, Figure S1: Viewing angle profile; Figure S2: 45° tilted SEM image of (*a*) only O_2_ plasma treatment and (*b*) only CHF_3_ plasma treatment, and (*c*) current density-EQE graph of OLEDs without and with nano-patterned/non-patterned MLA; Figure S3: Normalized spectrum with changing viewing angle; Figure S4: CIE’ 1931 color coordinate emission of fabricated OLEDs as a function of the viewing angle; Figure S5: 45° tilted SEM image according to O_2_ plasma and CHF_3_ plasma treatment time. (O_2_ time/CHF_3_ time); Table S1: Re-calculated EQE using viewing angle profile.
