# Supplementary material for: Enhanced Light Extraction from Organic Light-Emitting Diodes with Micro-Nano Hybrid Structure
Source: Nanomaterials (Basel). 2022 Apr 8;12(8):1266. doi: 10.3390/nano12081266 (PMC9031578; doi:10.3390/nano12081266)
Supplement: Supplementary file 1 [file nanomaterials-12-01266-s001.zip › nanomaterials-1636625-supplementary.pdf]

nanomaterials

*Supplementary Materials*

# Enhanced Light Extraction from Organic Light-Emitting Diodes with Micro-Nano Hybrid Structure

**Eun-Jeong Bae [1,2], Shin-Woo Kang [1,2], Geun-Su Choi [1], Eun-Bi Jang [1], Dong-Hyun Baek [3,*], Byeong-Kwon Ju [2,*] and Young-Wook Park [1,*]**

[1] Nano and Organic-Electronics Laboratory, Department of Display and Semiconductor Engineering, SunMoon University, Asan 31460, Korea; baeej2@sunmoon.ac.kr (E.-J.B.); newoosw@korea.ac.kr (S.-W.K.); crs4964@sunmoon.ac.kr (G.-S.C.); kksk0428@sunmoon.ac.kr (E.-B.J.)

[2] Display and Nanosystem Laboratory, Department of Electrical Engineering, Korea University, Seoul 02841, Korea

[3] Center for Next Generation Semiconductor Technology, Department of Display and Semiconductor Engineering, Sun Moon University, Asan 31460, Korea

* Correspondence: dhbaek@sunmoon.ac.kr (D.-H.B.); bkju@korea.ac.kr (B.-K.J.); zerook@sunmoon.ac.kr (Y.-W.P.)

## 1. EQE calculation using viewing profile

**Table S1.** Re-calculated EQE using viewing angle profile.

| **Sample $O_2$ time / $CHF_3$ time** | **EQE at 20mA/$cm^2$ ⓐ** | **Viewing angle intensity coverage between 0˚~90˚* ⓑ** | **Conversion factor compared to Lambertian profile** ⓒ** | **Recalculated EQE ⓓ=ⓐ*ⓒ** |
|---|---|---|---|---|
| Lambertian*** | | **53.7%** | 100% | 1.28 |
| reference | 1.13 | 60.8% | 113% | 1.28 |
| 0/0 | 1.55 | 54.3% | 101% | 1.57 |
| 100/100 | 1.62 | 58.7% | 109% | 1.77 |
| 100/400 | 1.55 | 59.2% | 110% | 1.71 |
| 400/100 | 1.57 | 60.3% | 112% | 1.76 |
| 400/400 | 1.5 | 60.3% | 112% | 1.68 |

* **Viewing angle intensity coverage, compare with I=1 at all angles between 0˚~90˚.**

** **ⓒ = ⓑ of sample / ⓑ of Lambertian profile**

*** **Calculated by Lambertian profile of $I=I_0\cos\Theta$, $I_0$= intensity at incident angle = 0˚.**

Although the EQE should be calculated using total luminous flux, it is hard to measure precisely on a lab-scale since it requires a large scale and highly expensive 3D automation measurement system or integral measurement system using a goniometer or integral spheres. So, the simple calculation using viewing angle profile is widely used and adopted in this work. It consists of the following procedures.

1) Measure the EL characteristics with perpendicular emission (incident angle = 0˚).
2) Calculation of the total luminous flux assuming the light source has Lambertian emission profile, and calculate the '$EQE_{assuming\ Lambertian}$'
3) Measure the viewing angle profile
4) Calculate the conversion factor compared to the Lambertian light source
5) Apply conversion factor to the previously calculated '$EQE_{assuming\ Lambertian}$'.
6) EQE = conversion factor * '$EQE_{assuming\ Lambertian}$'.

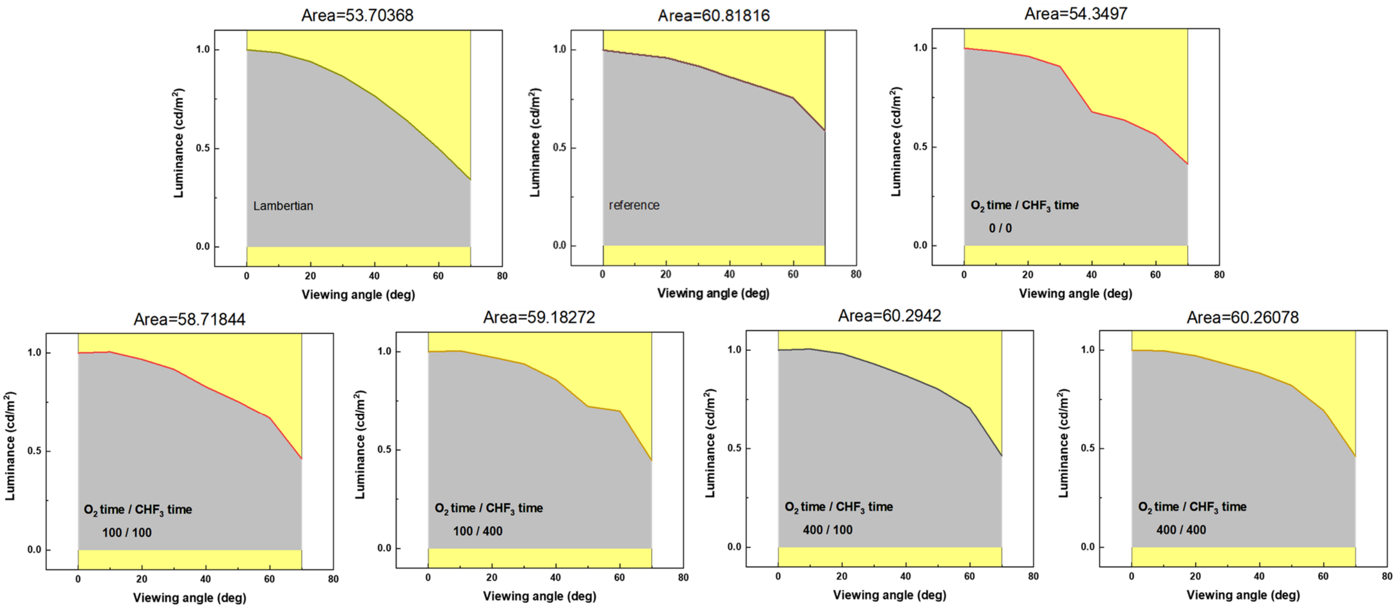


**Figure S1.** Viewing angle profile.

## 2. Light extraction characteristics according to $O_2$ plasma and $CHF_3$ plasma treatment

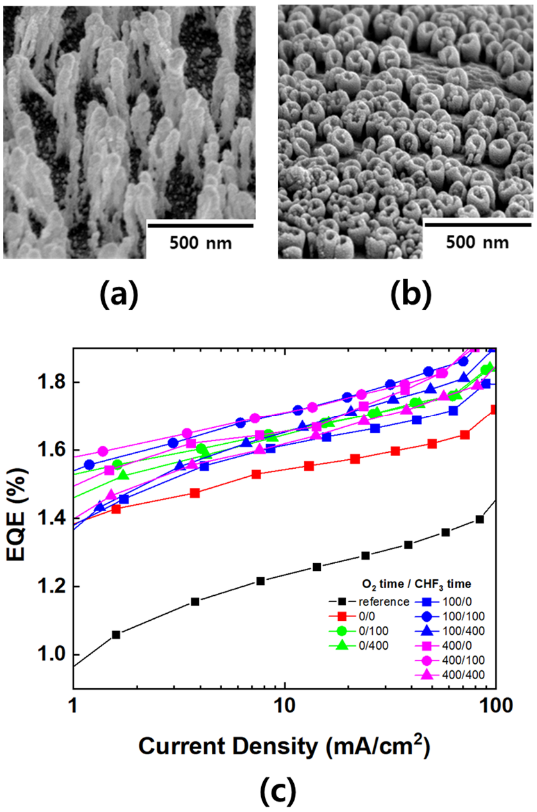


**Figure S2.** 45° tilted SEM image of (**a**) only $O_2$ plasma treatment and (**b**) only $CHF_3$ plasma treatment, and (**c**) current density-EQE graph of OLEDs without and with nano-patterned/non-patterned MLA.

The SEM images in Figure S2 (a) and (b) show the case where only $O_2$ plasma and only $CHF_3$ plasma were treated on the MLA surface, respectively. When only $O_2$ plasma was treated, the height of the nanorod was increased, whereas when only $CHF_3$ plasma was treated, the height of the nanorod was relatively low and wide. In Figure S2 (c), the overall EQE of blue and pink circle symbols was high. It is judged that the effect of $CHF_3$ plasma is greater than that of $O_2$ plasma on the efficiency characteristics. In addition, the efficiency was relatively higher when the combined plasma ($O_2$ + $CHF_3$) treatment was performed than when the $O_2$ plasma and $CHF_3$ plasma were treated alone. According to these results, it is necessary to process the complex plasma. Therefore, the light extraction structure was fabricated by first treating the $O_2$ plasma to increase the height of the nanopillars and then adjusting the width through $CHF_3$ plasma treatment.

## 3. Spectral and color coordinate characteristics according to viewing angle

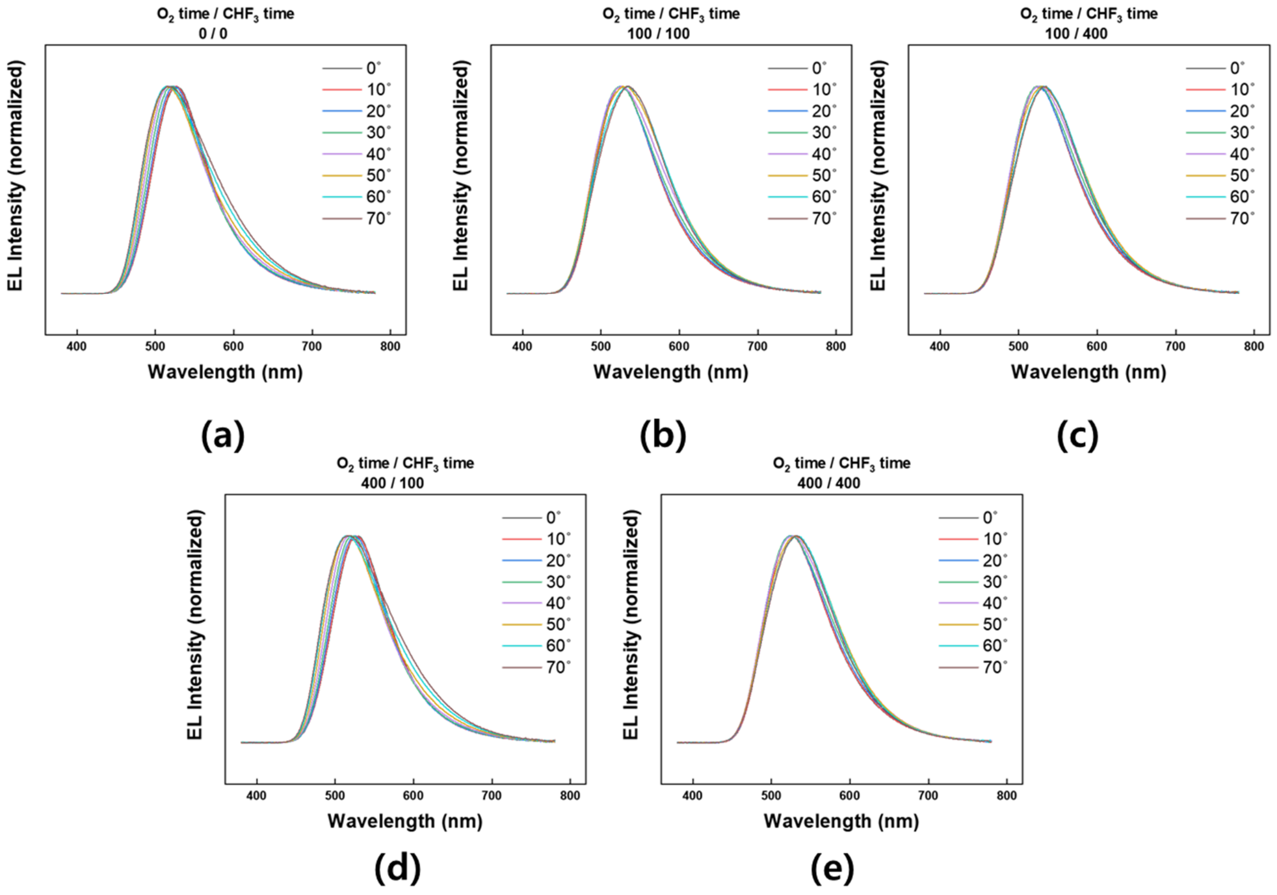


**Figure S3.** Normalized spectrum with changing viewing angle.

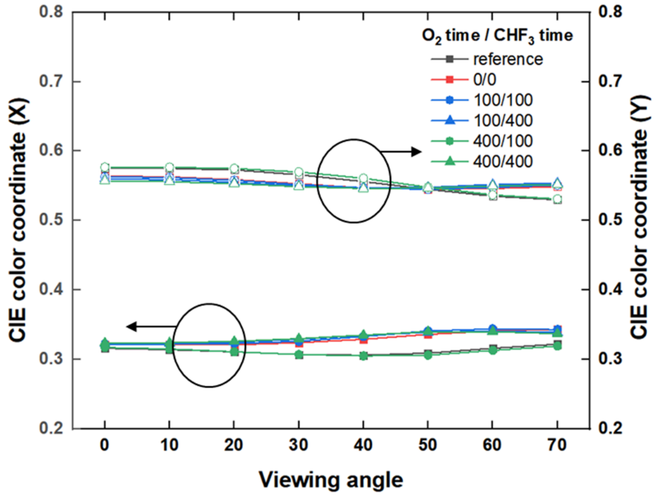


**Figure S4.** CIE′ 1931 color coordinate emission of fabricated OLEDs as a function of the viewing angle.

Figure S4 shows the CIE′ 1931 color coordinate emissions. The difference in CIE x and y of each device is less than 0.05 according to the change in viewing angle. It indicates that the micro-nano hybrid structure does not induce severe distortion in Figure S3.

## 4. Nanostructures formed by $O_2$ and $CHF_3$ plasma

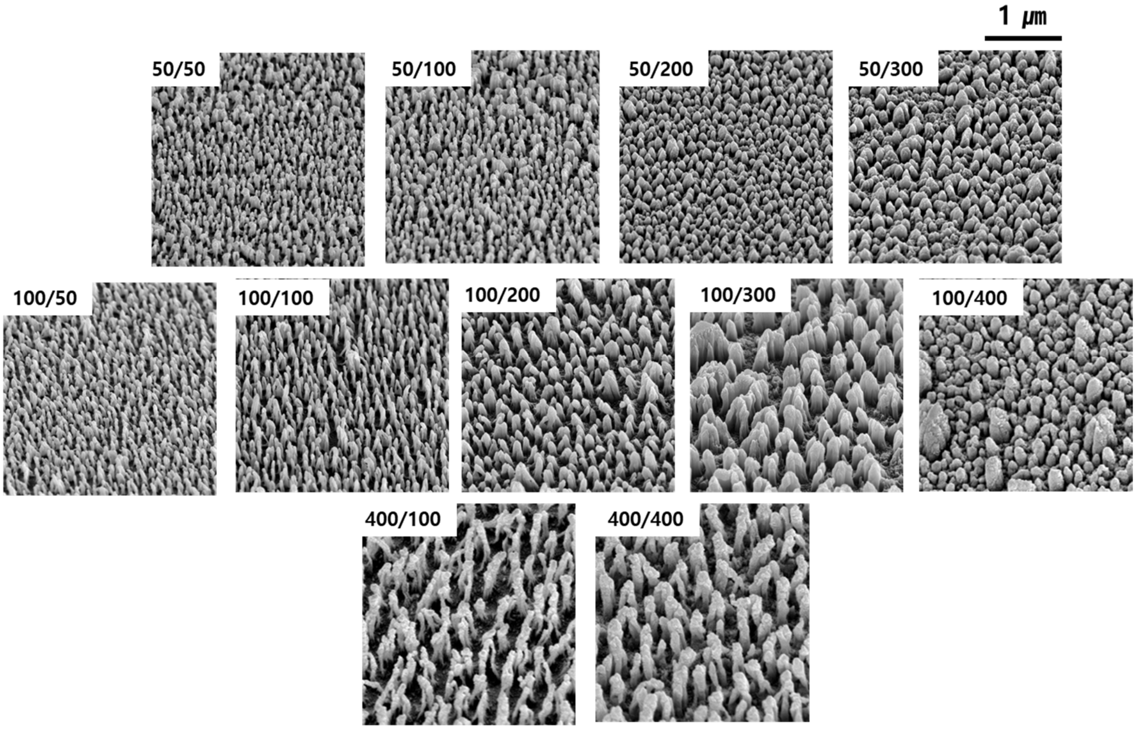


**Figure S5.** 45° tilted SEM image according to $O_2$ plasma and $CHF_3$ plasma treatment time.($O_2$ time / $CHF_3$ time).
